# Phenolic Compounds from *Ruta chalepensis* Var. *angustifolia* as Potential HMG-CoA Reductase Inhibitors: Bioactivity-Guided Fractionation and Preliminary Identification of Active Metabolites

**DOI:** 10.3390/molecules31193552

**Published:** 2026-10-05

**Authors:** Giselle Teresa-Martínez, Patricia Rodríguez-Castillo, Anayantzin Torres-Morales, Goretti Arvizu-Espinoza, Carmen Téllez-Pérez, Anaberta Cardador-Martínez, Maritza Alonzo-Macías

**Affiliations:** 1 Escuela de Ingeniería y Ciencias, Tecnologico de Monterrey, Epigmenio González 500, Querétaro 76130, Mexico; gis_teresa20@outlook.com (G.T.-M.); prcastillo@tec.mx (P.R.-C.); goretti@tec.mx (G.A.-E.); 2 Eco-Intensification of Agro-Industrial Eco-Processes, Laboratory of Engineering Science for Environment LaSIE-UMR-CNRS 7356, Institute Littoral Urbain Durable Intelligent, La Rochelle University, 17042 La Rochelle, France; ctellezperez@gmail.com

**Keywords:** HMG-CoA reductase inhibition, rutin, phenolic compounds, bioactive metabolites, hypercholesterolemia

## Abstract

*Ruta chalepensis* L. var. *angustifolia* (Pers.) Willk. (Rutaceae) has been traditionally associated with hypocholesterolemic effects, although the bioactive metabolites responsible for this activity remain largely unknown. This study aimed to fractionate the methanolic extract of *R. chalepensis* var. *angustifolia*, identify its major phenolic constituents, and evaluate their inhibitory activity against 3-hydroxy-3-methylglutaryl coenzyme A (HMG-CoA) reductase. The methanolic extract was fractionated by open column chromatography, and selected fractions were analyzed by thin layer chromatography and HPLC-DAD. Rutin was identified as one of the major flavonoids, while three chromatographically pure fractions corresponded to unidentified phenolic compounds. Their inhibitory activity was assessed using a commercial HMG-CoA reductase assay. Pravastatin showed 87.5% inhibition and served as the positive control. Among the isolated fractions, one unidentified phenolic fraction exhibited the highest inhibitory activity (66.0%), exceeding that of rutin (45.8%), whereas another fraction showed negligible activity (8.3%). These findings indicate that the HMG-CoA reductase inhibitory potential of *R. chalepensis* var. *angustifolia* cannot be attributed solely to rutin and suggest the presence of additional bioactive phenolic metabolites. Further structural characterization and mechanistic studies are required to identify the active compound and confirm its hypocholesterolemic potential in biological systems.

## 1. Introduction

Cholesterol is the primary sterol in mammals and plays a crucial role in various essential cellular functions. In addition to serving as a precursor for steroid hormones, bile acids, and oxysterols and contributing to the structural integrity of cell membranes, it regulates cell signaling through lipid rafts and the Hedgehog signaling pathway [1].

When cholesterol accumulates in the blood, the resulting hypercholesterolemia becomes one of the principal risk factors for atherosclerosis and cardiovascular disease (CVD) [2]. CVD, through myocardial infarction and stroke, remains the leading cause of death worldwide [3]. Moreover, because lipoproteins are central to atherogenesis, lipoprotein disorders are clinically important, and lowering cholesterol in affected patients reduces the incidence of cardiovascular events [4].

Cholesterol biosynthesis begins with acetyl-coenzyme A, which is generated in mitochondria and exported to the cytosol. There, acetyl-CoA and acetoacetyl-CoA are combined to form 3-hydroxy-3-methylglutaryl-CoA (HMG-CoA) by HMG-CoA synthase, which HMG-CoA reductase then reduces to mevalonate [5]. This reduction is the rate-limiting step of the pathway and therefore the main point of pharmacological control.

Statins exploit precisely this step. As competitive inhibitors of HMG-CoA reductase, they bind the enzyme’s active site with an affinity ten thousand times higher that of the natural substrate HMG-CoA, blocking the formation of mevalonate and thereby suppressing cholesterol synthesis in hepatocytes [6,7]. The consequent fall in intracellular cholesterol up-regulates hepatic LDL receptors, further lowering circulating LDL cholesterol [6,8].

Plant-derived compounds offer a complementary source of HMG-CoA reductase inhibitors. Extracts of the genus *Ruta* have been reported to possess antimicrobial, cytotoxic, anti-inflammatory, antioxidant, and hypocholesterolemic activities [9,10]; the specific metabolites responsible for these effects and their mechanisms of action remain largely undefined.

Phenolic compounds are the most abundant secondary metabolites in plants and are commonly grouped into flavonoids, phenolic acids, tannins, stilbenes, and lignans according to their carbon skeleton [11]. They display a broad spectrum of biological activities such as anti-oxidant, anti-inflammatory, and antihypertensive activities [12]. Flavonoids have been associated with a reduced risk of CVD [13,14].

Among the phenolic-rich botanical families, Rutaceae stands out for its aromatic essential oils and includes several perennial shrubs, of which *R. chalepensis* and *R. graveolens* are the most widespread [15,16]. Ruta graveolens and its constituents show a remarkable range of pharmacological activities, and the genus has been subject to research for conditions as diverse as memory impairment, depression, and cancer, as well as for its antibacterial, antifungal, and antiparasitic properties [10,17,18].

*Ruta chalepensis* L. var. *angustifolia* (Pers.) Willk. is a species with numerous medicinal properties [19,20], notably in the cardiovascular context, where it inhibits platelet aggregation [21]. Furthermore, within the genus, several cardiovascular activities have been documented; the aqueous extract of *R. montana* exerts vasorelaxant activity in isolated aortic rings and lowers blood pressure in hypertensive rats [22]. Its extracts additionally exhibit antimicrobial, anti-inflammatory, and cytotoxic activities, most of which are attributed to phenolic compounds [23,24,25,26]. Of particular relevance to the present work, the ethanolic extract of *R. chalepensis* var. *angustifolia* markedly reduced total cholesterol and LDL-C in hyperlipidemic rats, outperforming fenofibrate, an effect the authors attributed to downregulation of HMG-CoA reductase and upregulation of the LDL receptor [19]. That proposed mechanism has not, however, been verified at the enzyme level, nor have the specific metabolites responsible been identified. Among the phenolic constituents of the extract, rutin, a flavonoid glycoside reported in both methanolic and ethanolic *Ruta* extracts, has been shown to have biological activities including prevention of memory loss and anti-oxidant and anti-inflammatory effects [17,19,27].

On this basis, the present study aims to fractionate the methanolic extract of *R. chalepensis* var. *angustifolia*, identify its phenolic constituents, and evaluate their inhibitory activity against the HMG-CoA reductase enzyme.

## 2. Results and Discussion

### 2.1. Fraction Analysis by TLC and HPLC

The methanolic extract (ME) of *R. angustifolia* leaves was obtained with a yield of 0.23 ± 0.09 g extract/g dry plant (roughly 23%), comparable to the 25% reported for *R. chalepensis* var. *angustifolia* leaves by Al-Ismail, Al-Atewi [28], and within the range reported for methanolic extracts of the species harvested at different sites (12.4–20.1%) by Zohra, KanounKhedoudja [29]. The variation is attributable to the natural variability of the plant material since flavonoid and phenolic accumulation responds strongly to abiotic factors such as UV exposure, temperature, and water availability [30].

**Figure 1:**
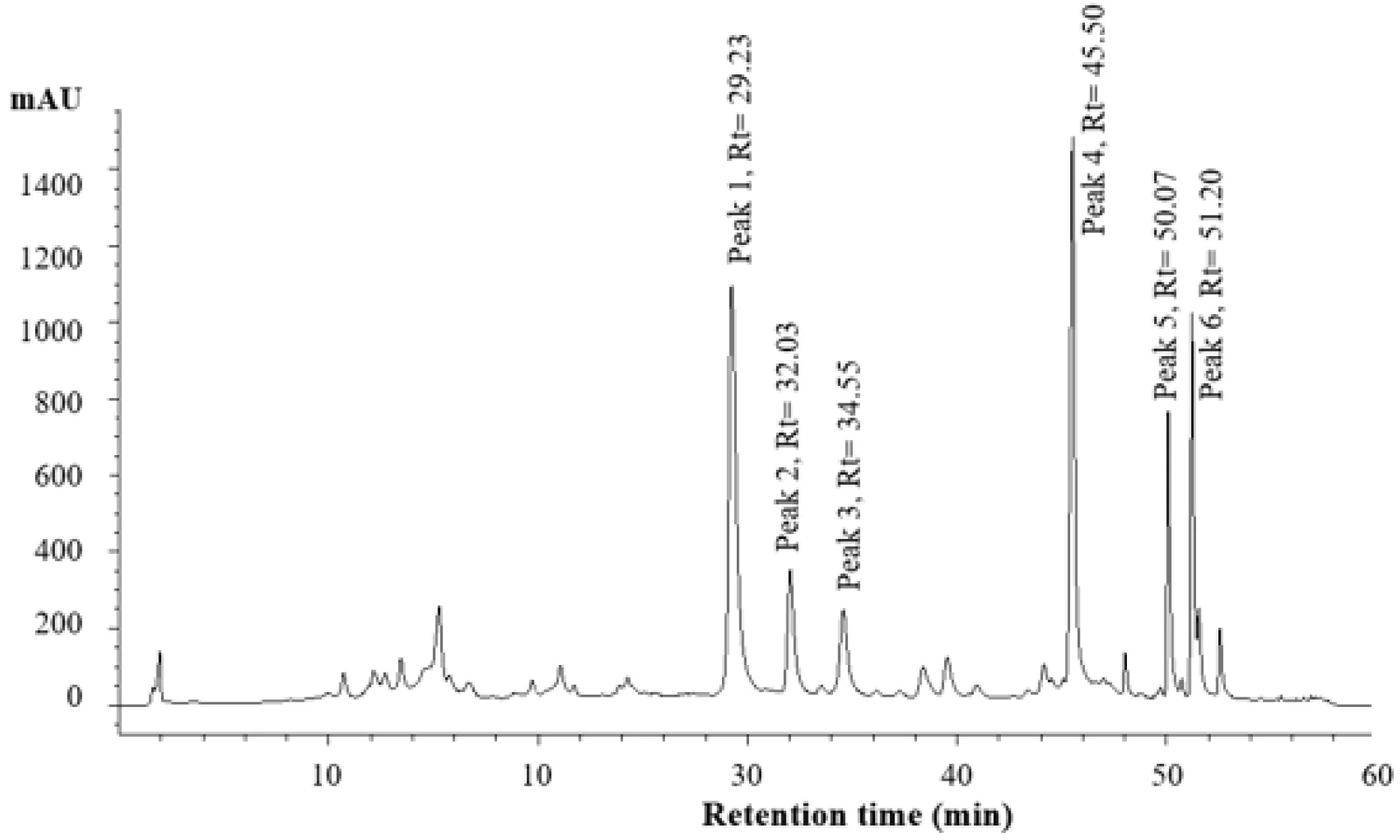
An HPLC-DAD chromatogram of the whole methanolic extract, showing the six principal peaks (peak 1 = rutin). λ = 280 nm.

**Figure 2:**
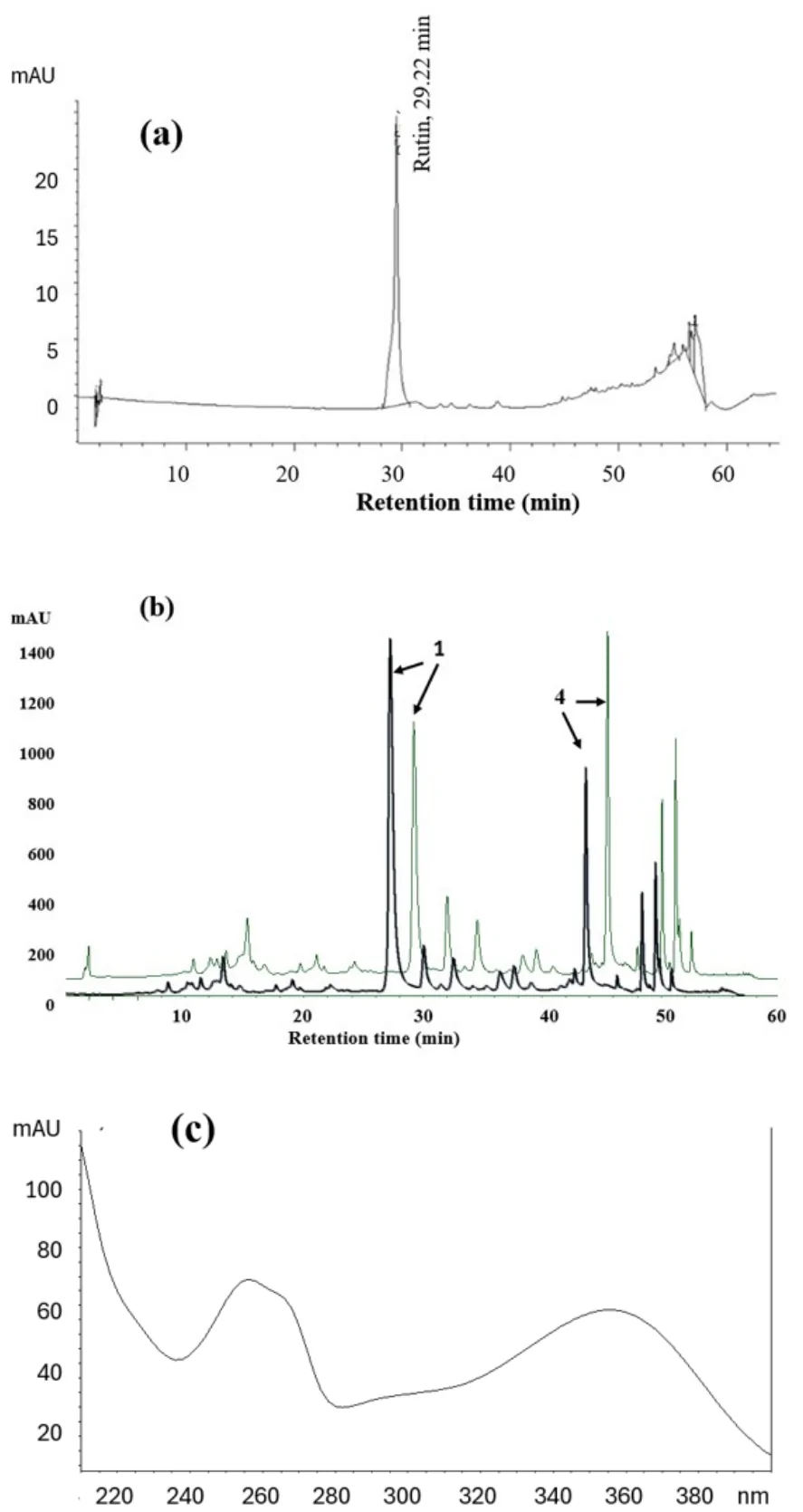
The identification of rutin. (**a**) A chromatogram of the rutin standard (Rt 29.22 min). (**b**) Co-elution (standard addition) comparison of the whole ME (green) overlaid with the ME spiked with rutin standard (black) Numbers 1 and 4 indicate the peak number according to elution time. (**c**) UV scan of rutin.

HPLC-DAD analysis of the whole extract resolved a flavonoid profile dominated by six principal peaks (Figure 1; Table 1). Peak 1, eluting at 29.23 min, coincides with the retention time of the rutin standard (Figure 2a). Its identity was confirmed by two independent criteria rather than retention time alone. First, in the unspiked extract, peak 4 was taller than peak 1, but upon spiking the extract with rutin standard, the height ratio inverted and peak 1 became dominant (Figure 2b). Furthermore, the UV scan of peak 1 reproduced the characteristic rutin maximal absorption at 256 and 354 nm (Figure 2c) [31].

Quantification was made against a rutin calibration curve (y = 29042x + 258.12; R^2^ = 0.9971); the six peaks are summarized in Table 1 as milligrams of rutin equivalents (RE) per gram of dry extract. Rutin (peak 1) and the constituent at 45.50 min (peak 4) were the two most abundant compounds, at 53.76 ± 2.71 and 42.08 ± 2.01 mg RE/g dry extract, respectively. That most of the flavonoid content resides in only two of the six peaks indicates that its bioactivity cannot be presumed to reside in rutin alone.

The rutin content measured sits within the broad range documented for the genus; in R. graveolens analyzed by Mokhtar et al., 2022, rutin was the single most abundant phenolic, accounting for 45.98% of the quantified polyphenols [32], and in *R. chalepensis* var. *angustifolia* it has been recovered as one of the principal flavonoids isolated by HPLC, most efficiently as a methanolic extract [33]. An earlier study of *R. chalepensis* var. *angustifolia* leaves reported a higher rutin content of 266.7 mg/g dry extract in the research by Loizzo et al., 2018 [34]. Because rutin is a product of secondary metabolism, such variation is expected, since its accumulation depends on species, plant part, origin, harvest season, and extraction solvent, and flavonoid biosynthesis responds to abiotic stressors such as UV exposure and temperature [29,30]. Solvent polarity is especially influential, with aqueous methanol being among the most effective media for rutin recovery [35].

The choice of a hydroalcoholic solvent reflects the polarity-driven behavior of phenolic compounds. Aqueous methanol recovers a broad span of the phenolic pool, whereas highly methoxylated, less polar forms partition preferentially into solvents such as ethyl acetate or chloroform [35]. The extract obtained was chemically heterogeneous, and therefore subsequently resolved on a normal-phase column eluted with a chloroform–ethyl acetate gradient of increasing polarity. The obtained phenolic compound-enriched fraction was analyzed by TLC methodology, where two complementary detection reagents were used.

Column effluent was monitored by TLC as a screening procedure to guide the pooling of consecutive fractions and their selection for chromatographic analysis. On this basis, this technique was not suitable for compound identification or purity assessment; accordingly, no chemical inference was drawn at this stage. The pooled fraction groups F25–F34, F63–F72, F111–F120 and F271–F280 were selected for HPLC analysis.

**Figure 3:**
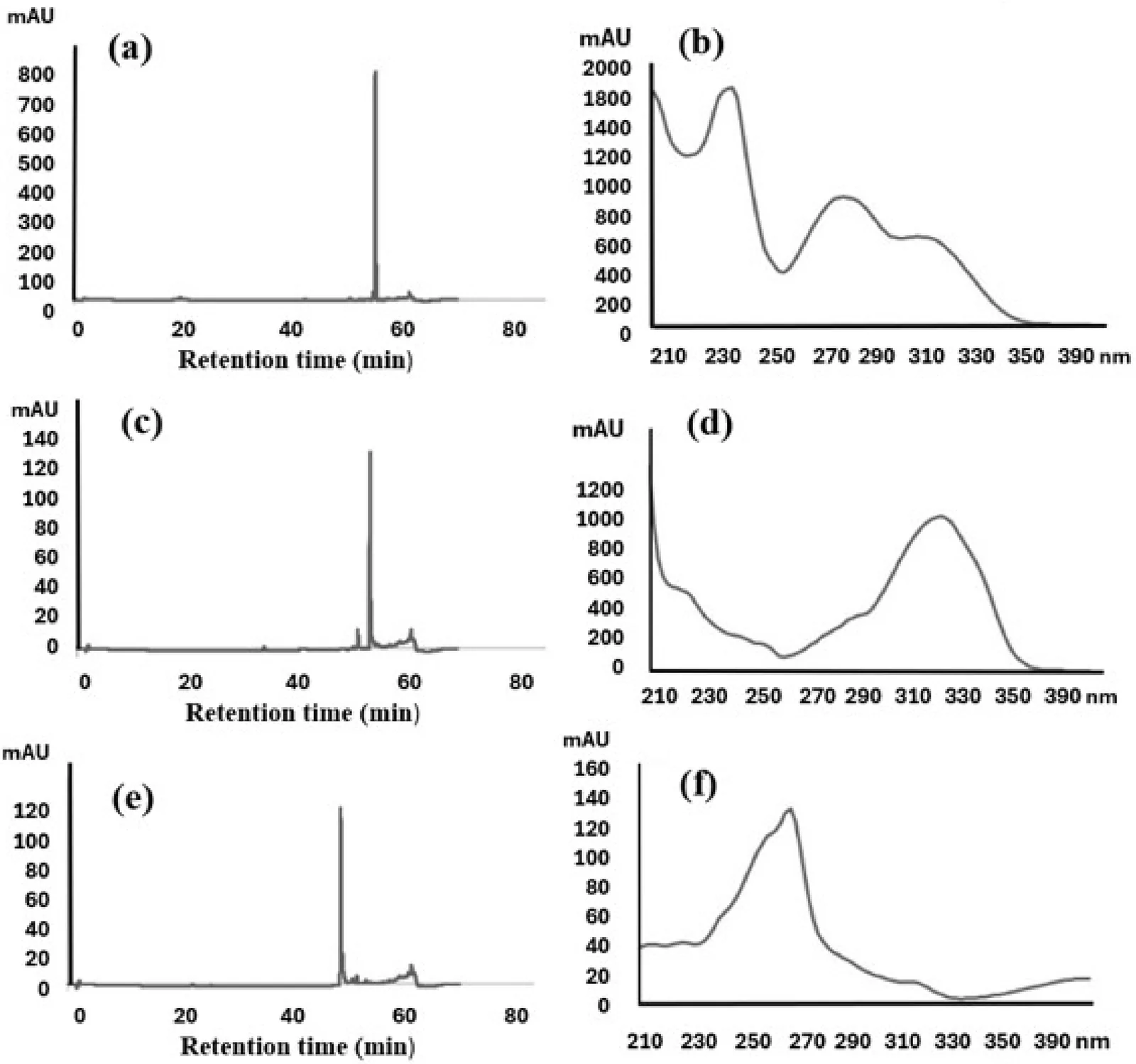
(**a**,**b**) Chromatogram and UV scan (210–400) nm of Fraction A. Rt = 51.19 min and Max Abs = 246 nm, 290 nm and 326 nm. (**c**,**d**) Chromatogram and UV scan (210–400) nm of Fraction B. Rt = 50.07 min and Max Abs = 336 nm. (**e**,**f**) Chromatogram and UV scan (210–400) nm of Fraction C. Rt = 45.35 min and Max Abs = 274 nm.

HPLC-DAD analysis of the selected fractions (Figure 3) showed that fractions F25–F34 (Fraction A), F111–F120 (Fraction B), and F271–F280 (Fraction C) each resolved into a single peak, indicating that the open column procedure isolated a chromatographically pure or near-pure fractions. None of the three peaks matched the standards screened. This can indicate that the isolated fractions are not among the common phenolic acids and flavonoids used as references, and may instead correspond to less-common phenolics, glycosylated derivatives, or other metabolite classes characteristic of the genus, which is notably rich in coumarins and furoquinoline alkaloids alongside its flavonoids [32].

The UV spectra nonetheless support a phenolic assignment when interpreted through the two-band absorption framework of flavonoid chromophores, in which band II (240–295 nm) arises from the benzoyl (A-ring) system and band I (300–380 nm) from the cinnamoyl (B-ring) system of more conjugated structures [31,36,37]. Fractions A and C displayed maximal absorbance near 280 nm (290 and 274 nm, respectively), consistent with a benzoyl-type phenolic chromophore. Fraction A additionally absorbed at 246 and 326 nm and Fraction B at 336 nm; these longer-wavelength bands fall in the band-I region and reflect the extended conjugation typical of flavones, flavonols and hydroxycinnamic-acid derivatives. Some phenolic acids such as 3-Caffeoylquinic acid have been identified in methanolic extracts of *R. chalepensis* with anti-oxidant and anti-inflammatory potential [19]. Fraction C, by contrast, showed only the 274 nm band with no band-I absorption, a pattern characteristic of flavonoids lacking A–B ring conjugation, such as flavanones, which show a strong band II near 270–295 nm and at most a weak band-I shoulder [31,36]. Moreover, flavonoids such as 3,3′,4′,5,7-Pentahydroxyflavone, 3-Methylquercetin and Myricetin have been identified in the methanolic extracts of *Ruta chalepensis* [19].

In the whole ME, a peak eluting at Rt = 29.23 min (Figure 1) coincided with the rutin standard (Rt = 29.22 min).

The recovery of a rutin-rich but chemically heterogeneous phenolic extract is coherent both with the polarity-driven behavior of these fractions and with the established phytochemistry of the genus, in which rutin is repeatedly identified as the principal flavonoid.

These assignments are based on DAD spectra alone and remain tentative; in the absence of structural data, the structures of the isolated constituents cannot be established; further studies are recommended in order to fill these gaps.

### 2.2. Inhibitory Activity of HMG-CoA Reductase

HMG-CoA reductase activity was evaluated indirectly by monitoring NADPH oxidation at 340 nm. Absorbance measurements were recorded every 20 s for 10 min, and each reaction was performed in triplicate. The assay included an uninhibited enzyme-activity control, pravastatin as the positive inhibitory control, rutin, the methanolic extract, and the chromatographically enriched fractions resuspended in methanol for HPLC. The fractions and extract were prepared at 4 mg/mL, and 1 µL of each sample was added to a final reaction volume of 200 µL, corresponding to a nominal assay concentration of approximately 20 µg/mL.

Pravastatin produced the highest apparent inhibition of HMG-CoA reductase, reaching 87.5%, thereby confirming the responsiveness of the enzymatic system. Among the plant-derived samples, the active fraction 120, designated as Fraction C, produced 66.0% inhibition, followed by rutin at 45.8%; on the other hand, Fraction A produced only 8.3% apparent inhibition.

Although Fraction A yielded a calculated inhibition value of 8.3%, its kinetic profile was comparable to that of the uninhibited enzyme control. The NADPH consumption rate therefore remained essentially unchanged in the presence of this fraction, indicating negligible or no detectable inhibition under the experimental conditions. Accordingly, the small percentage calculated for Fraction A should not be interpreted as evidence of biologically meaningful inhibitory activity, particularly in the absence of concentration–response data and variability estimates.

Rutin displayed moderate HMG-CoA reductase inhibition, supporting its possible contribution to the biological activity of the methanolic extract. Its presence in the extract had previously been supported by agreement between the retention time of the sample peak and the rutin standard, as well as by co-elution analysis. Nevertheless, rutin was less active than the unidentified fraction exhibiting 66.0% inhibition. This finding suggests that the HMG-CoA reductase inhibitory activity of *R. chalepensis* var. *angustifolia* cannot be attributed exclusively to rutin, despite its abundance in species of the genus *Ruta* [33,38]. Other constituents may possess greater intrinsic inhibitory activity or contribute to the extract’s overall activity through additive or synergistic interactions [39,40,41].

The enzymatic assay also revealed an important limitation in interpreting the methanolic extract and Fraction B. Both samples caused greater NADPH depletion than the uninhibited enzyme control. Since the assay estimates HMG-CoA reductase activity from the decrease in NADPH absorbance, this behavior cannot be interpreted as enzyme inhibition. Instead, it suggests that constituents in these samples may have directly oxidized NADPH or participated in enzyme-independent redox reactions. This response is attributed to the potential pro-oxidant behavior of some phenolic compounds, which may become relevant at elevated concentrations or in the presence of transition metals [42,43,44].

This observation is critical because redox-active compounds can interfere with NADPH-coupled enzymatic assays and generate misleading estimates of enzyme activity [42,45,46]. Future assays should include sample-specific controls containing each fraction or extract together with NADPH and assay buffer but without HMG-CoA reductase. Additional controls lacking substrate would help distinguish direct NADPH oxidation, intrinsic sample absorbance, precipitation, and other non-enzymatic effects from genuine modulation of HMG-CoA reductase.

The observed inhibition is pharmacologically relevant because HMG-CoA reductase catalyzes the conversion of HMG-CoA into mevalonate, a rate-limiting step in the endogenous cholesterol biosynthetic pathway. Inhibition of this enzyme reduces intracellular cholesterol synthesis and can activate sterol-sensitive regulatory pathways, thereby increasing hepatic LDL-receptor expression and promoting the removal of circulating LDL cholesterol [47,48,49].

**Figure 4:**
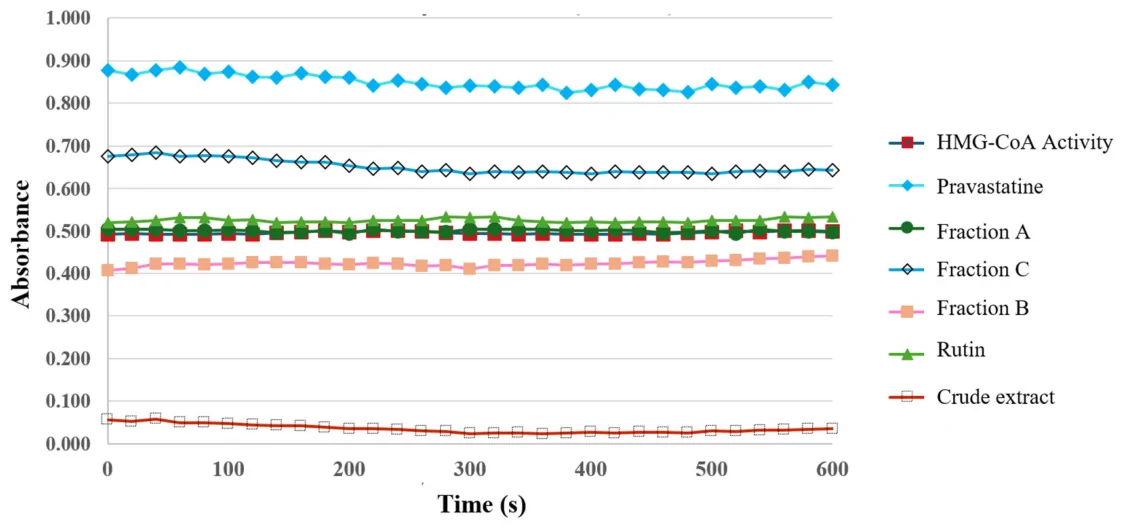
An enzymatic assay of the HMG-CoA reductase inhibition of Fractions A, B, C, rutin, and crude extract. Inhibitory effect: pravastatin 87.5%, Fraction C 66%, rutin 45.8%, and Fraction A 8.3%. The results are the mean of three repetitions, *n* = 3.

In the present study, we have evaluated HMG-CoA reductase in a cell-free system, and the results indicate enzymatic modulation under the specific assay conditions but do not demonstrate cholesterol-lowering activity in cells, animals, or humans. Translating this activity to a physiological setting depends on the absorption of the active constituents, whether sufficient hepatic exposure is achieved, whether the active constituents cross the hepatocyte membranes, and whether they maintain their inhibitory activity after biotransformation, none of which was assessed in this study. This limitation is particularly relevant for polyphenols, the permeability and bioavailability of which are highly affected by molecular size, polarity, glycosylation, and other structural characteristics. The passive membrane permeability of heavily hydroxylated or glycosylated polyphenols may be poor, but the absorption and cellular uptake of these compounds can be strongly influenced by deglycosylation and other metabolic transformations. In addition, absorbed polyphenols are often highly glucuronidated, sulfated, and methylated in the intestine and liver. Hepatocytes are likely to be exposed to conjugated metabolites rather than the parent fractions tested in the enzyme assay, and these metabolites may have reduced, retained, or otherwise changed inhibitory activity [50,51,52]. Thus, the present results should be considered indicative of biochemical inhibitory potential and a basis for further investigation, rather than evidence of therapeutic efficacy. Future studies should characterize intestinal permeability, hepatocyte uptake and intracellular exposure, and metabolic stability; determine activity of physiologically relevant metabolites; and confirm HMG-CoA reductase inhibition and cholesterol-lowering effects in hepatocyte-based and in vivo models. These results provide preliminary evidence that *R. chalepensis* var. *angustifolia* contains metabolites capable of inhibiting HMG-CoA reductase. It has already been proven in previous studies that plants rich in phenolic compounds can inhibit HMG-CoA reductase [53,54,55]. The unidentified active fraction, Fraction C, showing 66.0% inhibition, produced a higher percentage inhibition than rutin at the single concentration tested, and might be our primary candidate for further bioassay-guided purification and structural characterization. In contrast, Fraction A showed negligible activity, while the behavior of Fraction B and the crude extract was confounded by probable NADPH-related assay interference. The effect of Fraction A; Fraction C; Fraction B; rutin; pravastatin, a selective inhibitor of HMG-CoA reductase; and the crude extract is shown in Figure 4.

The biological relevance of the active fraction will depend on several properties not evaluated here, including solubility, chemical stability, cellular uptake, metabolism, bioavailability, cytotoxicity, and access to the catalytic domain of HMG-CoA reductase within the endoplasmic reticulum. The statistical analysis indicated that the responses obtained with rutin and the active fraction differed significantly from those of pravastatin according to Dunnett’s test at *p* < 0.05.

## 3. Materials and Methods

### 3.1. Plant Material

Fresh plant material was collected from Zacapu, Michoacán, Mexico (2041 msnm, 19°47′5.364″ N, 101°46′1.5″ O), in January 2019. The botanical identity was determined by a biologist, PhD. Víctor Manuel Rodríguez from the science and engineering school of Tecnológico de Monterrey. A voucher specimen comprising roots, stems, leaves, and flowers was taken from the same population, dried and herborized, and deposited at the Herbarium of Tecnológico de Monterrey Campus Querétaro (accession number TMA191001), where it was kept in darkness at 25 °C. The material was identified on the basis of morphological characteristics as *Ruta angustifolia* Pers. (Rutaceae), a taxon also treated under the synonym *R. chalepensis* L. var. *angustifolia* (Pers.) Willk.

### 3.2. Extract Preparation

The collected plants were dried in darkness at 25 °C for one week over paper sheets. After the leaves were collected, they were ground to powder in a coffee grinder (KrupsTM GX4100, Germany) and sieved to obtain fine flour (particle size of 625 μm). The powder was kept in the dark at room temperature (25 °C) until analysis.

The powdered leaves (50 g) were macerated in 700 mL of 70% methanol for three days [56,57]. The extract was filtered and dried by a rotary evaporator at 70 °C (Rotavapor^®^ R-215, BÜCHI Labortechnik AG, Germany). Finally, the dried extract (10 ± 0.8 g) was kept at −80 °C until analysis. This extract was called the methanol extract (ME).

### 3.3. Extract Fractionation by Column Chromatography

The fractionation of the extract was based on the protocol described by Arvizu-Espinosa, von Poser [58] with some modifications. The extract was separated by open column chromatography (37 cm × 4 cm), and 4 g of methanolic extract of *R. chalepensis* var. *angustifolia* leaves were adsorbed on 40 g of silica gel 60 (Merck^®^, 70–230 mesh) suspended in chloroform: ethyl acetate (9:1), which was used as the stationary phase. The methanolic extract was eluted using gradients of increasing polarity, starting with chloroform 90% (150 mL), 80% (200 mL), 70% (150 mL), 60% (150 mL), 50% (200 mL), 30% (150 mL) mixed with ethyl acetate, all the way up to pure ethyl acetate. Each fraction collected was 1 mL volume.

### 3.4. Fraction Analysis by Thin Layer Chromatography (TLC)

Fraction analysis was performed by thin layer chromatography (TLC). Aliquots of each fraction (5 µL) were applied onto silica gel 60 F_254_ chromatographic plates (5 × 5 cm; Merck, Darmstadt, Germany). The plates were developed for 10 min using chloroform/ethyl acetate (70:30, *v*/*v*) as the mobile phase. After development, the plates were derivatized using either p-anisaldehyde–sulfuric acid reagent, prepared with methanol/acetic acid/sulfuric acid (85:10:5, *v*/*v*/*v*) supplemented with 0.5 mL of p-anisaldehyde, or aluminum chloride solution (1 g/100 mL ethanol). Plates treated with p-anisaldehyde–sulfuric acid reagent were heated at 100 °C to promote color development. In contrast, plates treated with aluminum chloride were visualized under ultraviolet light at 365 nm to detect yellow fluorescence, as described by Gerlach, Stoschek [59].

### 3.5. Fraction Analysis by High-Performance Liquid Chromatography (HPLC)

Fractions selected during TLC screening were further analyzed by high-performance liquid chromatography (HPLC) using an Agilent 1100 system equipped with a diode array detector (DAD) (Agilent Technologies, Palo Alto, CA, USA). Chromatographic separation was performed on an Eclipse XDB-C18 column (5 µm, 4.6 × 150 mm; Agilent Technologies, USA). The column oven temperature was maintained at 25 °C.

Before injection, fractions were redissolved in HPLC-grade methanol. The reconstituted samples were filtered through a 0.45 µm syringe filter before analysis, and the injection volume was 20 µL.

Gradient elution was carried out using 0.01 M H_3_PO_4_ as solvent A and methanol as solvent B. The gradient program was as follows: 5% A at 0 min, 40% A at 25 min, 50% A at 40 min, 70% A at 45 min, 80% A at 50 min, and 100% A at 55 min. The flow rate was set at 1 mL/min, and detection was performed at 245 nm using the DAD.

Calibration curves were prepared using standard solutions at concentrations ranging from 0 to 0.1 mg/mL. The phenolic compounds used as analytical standards were gallic acid, caffeic acid, chlorogenic acid, (+)-catechin, 2-hydroxycinnamic acid, 4-hydroxybenzoic acid, 4-hydroxy-3-methoxycinnamic acid, p-coumaric acid, syringic acid, sinapic acid, apigenin, 3,4-dihydroxybenzoic acid, rutin, and quercetin. The calibration curves of the standards (0–0.1 mg/mL) were used.

### 3.6. Inhibition of HMGCoA Reductase

The inhibitory effect of the fractions on HMG-CoA reductase activity was evaluated spectrophotometrically by monitoring NADPH oxidation at 340 nm and 37 °C, using a commercial HMG-CoA Reductase Activity/Inhibitor Screening Kit (Catalogue number CS1090-1Kt, Sigma-Aldrich Química, S.L., Toluca, Mexico), following the manufacturer’s instructions; no pre-incubation was performed. Brief reactions were performed in a final volume of 200 μL (96-well plate), containing 4 μL NADPH, 12 μL HMG-CoA, and 2 μL HMGR, with 1 × assay buffer adjusted to the final volume. Inhibition reactions additionally contained 1 μL pravastatin (100 µM), whereas blanks contained no enzyme. The assay buffer was pre-equilibrated to 37 °C before sequential addition of pravastatin, where applicable; NADPH; and HMG-CoA. Reactions were initiated by adding HMGR and mixing thoroughly; microplates were shaken vigorously for 10 s before the first measurement. Absorbance was recorded immediately at 20 s intervals for up to 10 min. All reactions were performed in triplicate.

### 3.7. Statistical Analysis

All statistical analyses were performed using Minitab software, version 19, for Windows. Data were analyzed by one-way or two-way analysis of variance (ANOVA), as appropriate. When significant differences were detected, Dunnett’s post hoc test was applied to compare treatments against the control group. Differences were considered statistically significant at *p* < 0.05.

## 4. Conclusions

The methanolic extract of *R. chalepensis* var. *angustifolia* leaves proved to be a flavonoid-rich but chemically heterogeneous matrix, resolving by HPLC-DAD into six principal peaks. Rutin was identified as the earliest eluting of these (peak 1) on the combined evidence of retention-time coincidence, a standard-addition co-elution experiment, and UV spectral matching, and it ranked among the two most abundant constituents of the profile.

Open column fractionation guided by TLC color screening, followed by HPLC verification, yielded three chromatographically pure constituents (Fractions A, B and C), each corresponding to a distinct peak of the extract profile. None matched the phenolic-acid or flavonoid standards available, so their identities remain tentative; the UV spectra nonetheless place them in different phenolic subclasses. Definitive structural assignment will require further analysis to properly identify these fractions.

The ME of *R. chalepensis* var. *angustifolia* contained phenolic compounds, such as rutin, that can inhibit HMG-CoA reductase; however, more studies are needed to support its hypocholesterolemic effect. The crude extract and the B fraction induced NADPH oxidation, which may have distorted the results. In addition, another compound with a stronger inhibitory effect on this enzyme has been identified; more studies are needed to identify it. Future studies should establish full concentration–response curves, calculate IC_50_ values, determine the mode of inhibition via enzyme kinetic analysis, and confirm activity using orthogonal assays that directly quantify mevalonate formation or substrate conversion. Subsequent experiments in hepatic cell models should assess intracellular cholesterol synthesis, LDL-receptor expression, cytotoxicity, and cellular bioavailability before a hypocholesterolemic effect can be proposed.

## Figures and Tables

**Table 1:** The retention times and rutin equivalent content of the six principal peaks of the *R. angustifolia* methanolic extract.

Peak	Rt (min)	Content (mg RE/g Dry Extract)
1 (rutin)	29.23	53.76 ± 2.71
2	32.03	13.09 ± 0.22
3	34.55	10.47 ± 0.59
4	45.50	42.08 ± 2.01
5	50.07	14.81 ± 1.35
6	51.20	18.76 ± 1.95

## Data Availability

The original contributions presented in this study are included in the article. Further inquiries can be directed to the corresponding authors.

## Abbreviations

The following abbreviations are used in this manuscript:

HMG-CoA	3-hydroxy-3-methyl-glutaryl-coenzyme A reductase
HPLC-DAD	High-performance liquid chromatography–diode array detector
TLC	Thin layer chromatography
